# Developing a Relative Humidity Correction for Low-Cost Sensors Measuring Ambient Particulate Matter

**DOI:** 10.3390/s18092790

**Published:** 2018-08-24

**Authors:** Andrea Di Antonio, Olalekan A. M. Popoola, Bin Ouyang, John Saffell, Roderic L. Jones

**Affiliations:** 1 Centre for Atmospheric Science, Department of Chemistry, University of Cambridge, Lensfield Road, Cambridge CB2 1EW, UK; oamp2@cam.ac.uk (O.A.M.P.); bo237@cam.ac.uk (B.O.); rlj1001@cam.ac.uk (R.L.J.); 2 Alphasense Ltd., Sensor Technology House, 300 Avenue West, Skyline 120, Great Notley, Essex CM77 7AA, UK; jrs@alphasense.com

**Keywords:** air pollution, environmental monitoring, low cost sensors, particulate matter, relative humidity correction

## Abstract

There is increasing concern about the health impacts of ambient Particulate Matter (PM) exposure. Traditional monitoring networks, because of their sparseness, cannot provide sufficient spatial-temporal measurements characteristic of ambient PM. Recent studies have shown portable low-cost devices (e.g., optical particle counters, OPCs) can help address this issue; however, their application under ambient conditions can be affected by high relative humidity (*RH*) conditions. Here, we show how, by exploiting the measured particle size distribution information rather than PM as has been suggested elsewhere, a correction can be derived which not only significantly improves sensor performance but which also retains fundamental information on particle composition. A particle size distribution–based correction algorithm, founded on κ-Köhler theory, was developed to account for the influence of *RH* on sensor measurements. The application of the correction algorithm, which assumed physically reasonable κ values, resulted in a significant improvement, with the overestimation of PM measurements reduced from a factor of ~5 before correction to 1.05 after correction. We conclude that a correction based on particle size distribution, rather than PM mass, is required to properly account for *RH* effects and enable low cost optical PM sensors to provide reliable ambient PM measurements.

## 1. Introduction

There has been a growing interest in air quality monitoring in recent years with a large number of epidemiological studies demonstrating a link between human health diseases and air pollution (e.g., [1,2,3,4]). Of particular interest for health impacts is the measurement of PM concentrations [5]. There are standard limits for exposure to particle with a mean aerodynamic diameter less than 10 μm (PM_10_) and 2.5 μm (PM_2.5_) [5,6], although some studies also highlight the importance of exposure to smaller particles (e.g., PM_1_) [7,8].

Concentrations of particulate mass are generally highly structured both spatially and temporally, and thus, personal exposure to air pollution can differ significantly even on the street scale [9,10]. For this reason, there have been numerous attempts at producing low-cost portable PM sensors to create monitoring networks with much higher spatial resolution [11,12,13] or for personal monitoring [14]. Here we consider low-cost OPCs which use light scattering to determine the size and number concentration of particles which are then, making various assumptions, converted into mass concentration in the form of PM_1_, PM_2.5_ and PM_10_.

Water vapour can condense on aerosol particles, making them grow hygroscopically under high *RH* conditions [15]. To correct for this effect, reference instruments are usually equipped with drying systems which remove water from particles before measurement. Many low-cost OPCs do not include such drying processes, with the result that particle sizes can be overestimated at high *RH*, resulting in PM values are then enhanced relative to reference measurements. A recent study [16] has proposed an *RH* dependent correction factor to be applied to PM data to account for such high *RH* effects. This factor was determined using a statistical approach where PM measurements derived from an Alphasense OPC-N2 were fitted to TEOM reference instrument measurements using a κ-Köhler [17] type correction approach to determine an optimal average κ value for the period examined. Their approach, however, while statistically appealing, is in fact unphysical in that the application of a correction factor to the derived PM values is implicitly, even if not stated, equivalent to a uniform reduction in particle number concentration throughout the whole particle size spectrum. In reality, on dehydration, particles would reduce in size, not in number, thus affecting the derived PM in ways which now would depend on the detailed particle size spectrum. The approach we describe accounts in full for this shift in size.

**Figure 1:**
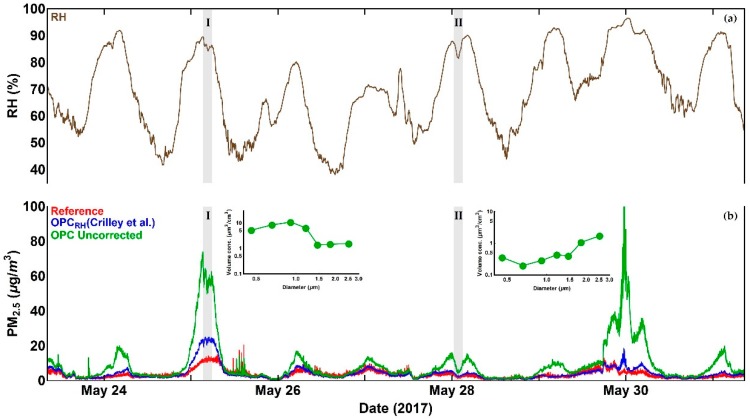
Time series of Alphasense OPC-N2 PM_2.5_ measurements compared with reference data: (**a**) Time series of relative humidity; (**b**) Time series of Alphasense OPC-N2 PM_2.5_ measurements, before and after the application of the Crilley et al. correction factor, in comparison with reference data. The two inserts show volume distribution profiles measured by the uncorrected OPC-N2 for periods I and II. The overall measurement period consists of seven consecutive days data between 23 May and 31 May 2017. See text for discussion. (PM: particulate matter).

We illustrate these effects by considering a series of PM measurements from an Alphasense OPC-N2 and a Palas Fidas 200 S (certified PM reference instrument) obtained for seven days in May 2017. PM_2.5_ measurements for this period, before and after the application of the correction factor proposed in [16], are presented, together with reference data, in Figure 1.

**Figure 2:**
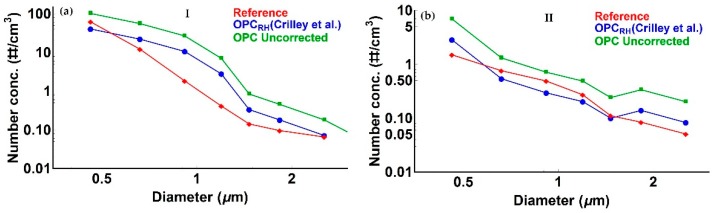
Particle size distributions for (**a**) period I in Figure 1 and (**b**) period II in Figure 1. Uncorrected Alphasense OPC-N2 particle size distributions are shown in green. Particle size distributions after application of the Crilley et al. correction factor (assumed to be constant across the size spectrum) are shown in blue, and reference data in red. See text for discussion.

As is evident from Figure 1 and as expected, there are significant enhancements in uncorrected Alphasense OPC-N2 PM_2.5_ readings relative to the reference measurements associated with high *RH* periods, although this is not always the case (i.e., period II in Figure 1). Application of the Crilley et al. correction factor [16] improves the Alphasense OPC-N2 derived PM compared to the reference data. Nevertheless, there are multiple periods (e.g., period I in Figure 1) where significant discrepancies remain. Those periods highlight the limitation of the approach presented in [16], when the particle number concentration distribution is relatively unstructured (i.e., period II in Figure 1 and Figure 2). Under such circumstances, a shift toward smaller sizes is broadly equivalent to scaling the number concentration down by a constant factor, as illustrated in Figure 2b, where the Crilley et al. correction produces tolerable agreement with the reference dried particle distribution, so that the reference and corrected PM_2.5_ values broadly agree. However, when the size distribution shows significant structure (e.g., period I in Figure 1), the approach presented in [16] fails to reproduce the reference dried particle size distribution, and PM values from the corrected OPC particle size spectrum are significantly overestimated.

The aim of this paper is to introduce an improved correction algorithm where *RH* effects can be better described by considering the detailed particle size profile. This algorithm accounts for the *RH* effect on the number concentration measurements of OPCs to ensure the correct PM values are calculated and, by retaining particle physical properties (κ values appropriate to specific chemical compositions), can be used to retrieve information about particles hygroscopicity and, in turn, their chemical composition.

## 2. Materials and Methods

### 2.1. Instrumentation

#### 2.1.1. Alphasense OPC-N2

The Alphasense OPC-N2 is a low-cost portable sensor manufactured by Alphasense Ltd., in Great Notley, UK. It uses light scattering to measure size speciated particle number concentrations, which are then converted in mass concentration in the form of PM_1_, PM_2.5_ and PM_10_. Particles passing through the sampling volume scatter incident laser light, which is then detected by a photo detector. Based on the amount of scattered light, particle size and number concentration are both determined. Measured particle size range, dimensions, and operational settings of the instrument are presented in Table 1. 

#### 2.1.2. Palas Fidas 200 S

Palas Fidas 200 S is an EN 16450 certified static PM measurement instrument [19] manufactured by Palas GmbH in Karlsruhe, Germany. It also uses light scattering to measure particle size and particle number concentrations which are again converted into PM_1_, PM_2.5_ and PM_10_. However, in this case, a drying system, the Intelligent Aerosol Drying System (IADS), is used to remove water from particles before measurement. Size range, dimensions, and operational settings of Palas Fidas 200 S are presented in Table 2. Unlike the Alphasense OPC-N2, this instrument uses a white LED laser which enables the detection of particles with a diameter as small as 0.18 μm (see Table 2).

### 2.2. Study Area

**Figure 3:**
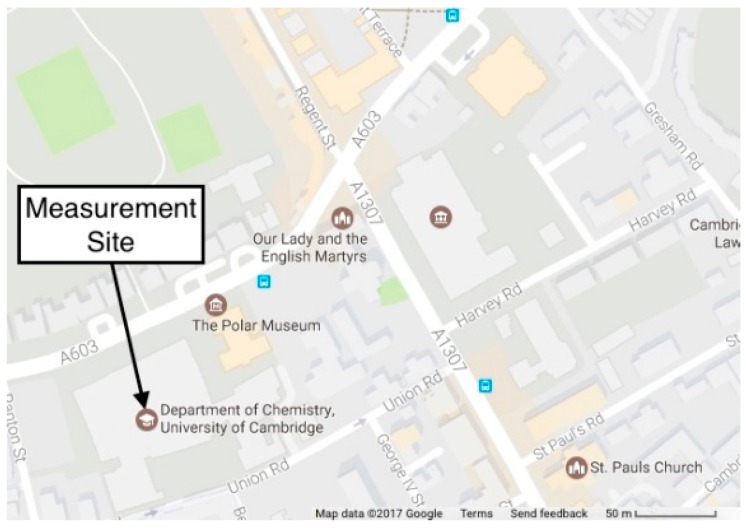
Map showing the location of measurement site in Cambridge, UK. Courtesy of Google Maps.

The location used for this study was the air quality and greenhouse gas monitoring station of the Centre for Atmospheric Science, University of Cambridge, UK. The measurement site is located on the roof of Chemistry Department at a height of 22 m above street level (52°11′52.4″ N, 0°07′31.9″ E). The site is close to a busy road junction in central Cambridge, as shown in Figure 3.

Alphasense OPC-N2 sensors were located in a waterproof shelter at the same height of the Palas Fidas 200 S instrument and within a distance of less than 1 m. The trials were initially performed with a single Alphasense OPC-N2 (24–31 May 2017), and then, for a more extended period (17 December 2017–16 January 2018), with two Alphasense OPC-N2 OPCs.

### 2.3. Data Processing

#### 2.3.1. Data Redistribution

**Figure 4:**
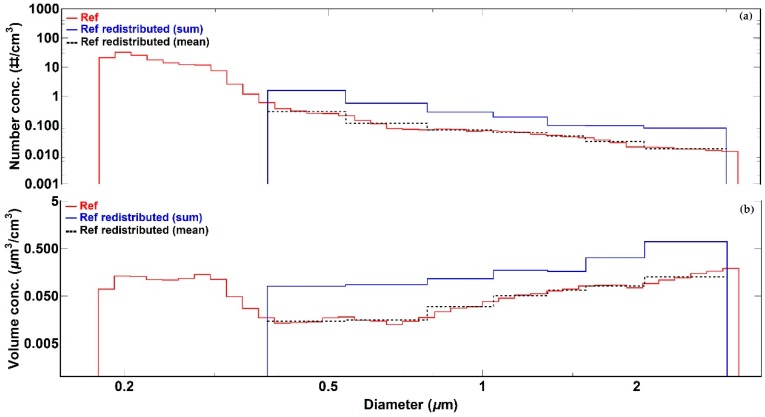
Illustration of Palas Fidas 200 S measurements redistributed into the Alphasense OPC-N2 size bins for all bins contributing to PM_2.5_ for (**a**) number concentration data and (**b**) volume concentration data. The black dashed lines represent the average Palas Fidas 200 S measurements in each Alphasense size bin.

Inspection of Table 1 and Table 2 indicates that the Alphasense OPC-N2 and the reference Palas Fidas 200 S instruments have different size ranges of particle measurement with different numbers of discrete measurement bins within which individual particles are summed (16 and 64, respectively). To ensure an appropriate comparison of OPC measurements, the reference particle number concentration values were transposed on to the Alphasense OPC-N2 size range and size bins. To do so, the fraction of each reference bin in each Alphasense OPC-N2 bin range was determined. To account for the case when only the lower boundary of the reference size bin sat within an Alphasense OPC-N2 size bin, the fraction was calculated as

(1) $$f_{low}=\frac{b_{upp}-b_{low}^{ref}}{b_{upp}^{ref}-b_{low}^{ref}},$$

where b is the Alphasense OPC-N2 bin, $b^{ref}$ is the reference bin, and the difference $b_{upp}^{ref}-b_{low}^{ref}$ is the width of the reference size bin. For the equivalent case when only the upper boundary of reference size bin sat within an Alphasense OPC-N2 size bin, the fraction was calculated as

(2) $$f_{upp}=\frac{b_{upp}^{ref}-b_{low}}{b_{upp}^{ref}-b_{low}^{ref}},$$

where b is the Alphasense OPC-N2 bin, $b^{ref}$ is the reference bin, and the difference $b_{upp}^{ref}-b_{low}^{ref}$ is the width of the reference bin. The new number concentration values for the reference data in the Alphasense OPC-N2 bins were given by the sum of all the calculated fractions within each Alphasense OPC-N2 size bins, multiplied by the associated number concentration value, as presented in Figure 4.

The advantage of using this redistribution technique is that it ensures an appropriate comparison of individual size bin data between Alphasense OPC-N2 sensors and the reference instrument. In this study, therefore, information about particle concentration in the 0.18–0.38 μm range is not used as the Alphasense OPC-N2 particle diameter detection limit is 0.38 μm (see Table 1).

#### 2.3.2. Number Concentration to Mass Conversion 

Both instruments used in this study utilise light scattering to determine particle size and particle number concentration. The conversion from particle number concentration to particle mass concentration in form of PM_1_, PM_2.5_ and PM_10_ is completed internally to each instrument. Particles are assumed to be spheres with uniform shape and density. In this work, the following equations were therefore used to convert particle concentration values to mass concentration:

(3) $$V_{i}=\frac{\pi }{6}\cdot {\left(D_{i}\right)}^{3},$$

(4) $$M_{i}=\rho \cdot V_{i},$$

(5) $$PM=\underset{i}{\sum }n_{i}\cdot V_{i}\cdot \rho ,$$

where $n_{i}$ is the particle number concentration value for the *i*th bin, $D_{i}$ is the mean diameter of the *i*th bin, $V_{i}$ is the particle volume in the *i*th bin, ρ is the particle density, and *i* is the bin number, spanning 1 to 16 (see Table 1). The particle density applied across all the bins from the Alphasense OPC-N2 is 1.65 g cm^−3^. Hence, for consistency, we used this value to calculate mass concentration values for both the OPCs and the reference instrument. PM_1_, expressed in μg m^−3^, was calculated via Equation (5) using bins 1–4. Equally, PM_2.5_ (μg m^−3^) was calculated via Equation (5) using bins 1–7. Penetration curves [20] are normally then used to convert the derived mass spectra to the appropriate PM values. However, for clarity, in the comparison of size and volume spectra for the different instruments, this step has not been applied.

#### 2.3.3. RH Correction Algorithm 

**Figure 5:**
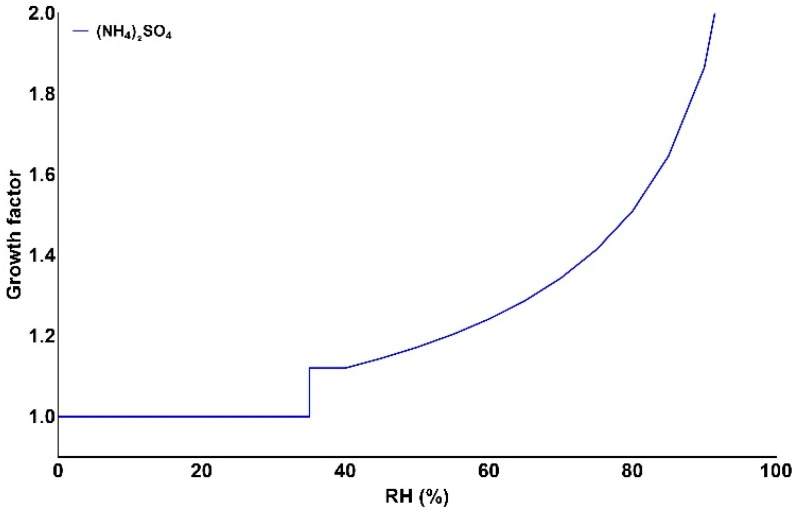
Growth factor curve as function of relative humidity (*RH*) for ammonium sulphate. The discontinuity represents the efflorescence point of the compound [27].

The correction algorithm presented in this study is based on the changes in particle size due to the water uptake. To quantify this effect, a hygroscopic growth factor was used [21].

(6) $$g\left(RH\right)=\frac{D_{wet}\left(RH\right)}{D_{dry}},$$

where $D_{dry}$ is the diameter of the dry particle and $D_{wet}\left(RH\right)$ is the diameter of the particle at a given *RH* value. Using the κ-Köhler theory [17], a single parameter relationship can be used to express the *RH* dependence of (6) as follows [22]:

(7) $$g\left(RH\right)={\left(1+\kappa \cdot \frac{RH}{100-RH}\right)}^{\frac{1}{3}},$$

where κ is a parameter that describes the degree of hygroscopicity of a particle, dependent on particle composition, and *RH* is the relative humidity [21]. To estimate the hygroscopic growth factor, knowledge of particle composition is required. This study focuses on urban environments, where the dominant aerosol inorganic components are sulphates and nitrates [22,23]. κ values for ammonium sulphate and ammonium nitrate are 0.61 and 0.67 [24,25]. As reported in [26], the κ value for a mixture of organic and inorganic compounds in polluted environments (MIXPO), such as urban, is $\kappa_{MIXPO}=0.62$ [26]. This mixture is a more realistic representation of the complex urban aerosol chemical composition. However, there is no information about the efflorescence point of this mixture. This implies that it is not possible to determine the *RH* value for which particles are no longer absorbing water. In contrast, the efflorescence point of ammonium sulphate is known to be at *RH* = 35% [27]. Considering the small difference in hygroscopicity of the two compounds and the information about the efflorescence point, we assumed particulate matter to be composed only of ammonium sulphate (κ=0.61). Under this assumption, g(RH) values for 0–100% *RH* range, at 5% intervals, were calculated and presented in Figure 5. 

From the knowledge of the hygroscopic growth factor g(RH) and measurement of particle diameters at different *RH* values, dry diameters were calculated rearranging (6) and (7) as

(8) $$D_{dry}=\frac{D_{wet}\left(RH\right)}{{\left(1+\kappa \cdot \frac{RH}{100-RH}\right)}^{\frac{1}{3}}}.$$

The particle number concentration values, while remaining unaltered, are now associated with new, *RH*-corrected, size bin limits which are shifted to smaller sizes according to Equation (8). As the fundamental comparison is performed using the Alphasense bin size range, we redistribute the *RH* corrected particle number concentration values to the original bin size ranges using equations analogous to Equations (1) and (2) but now substituting $b^{ref}$ with $b^{cor}$.

#### 2.3.4. RH Correction Statistical Validation

To quantify the improvement of the correction method, a statistical analysis was performed, and the following parameters were calculated for PM_1_ and PM_2.5_ data: mean value of measurements, standard deviation (SD), root-mean-square error (RMSE), gradient, and coefficient of determination *R*^2^. The gradient of the scatterplot and coefficient of determination *R*^2^ were calculated assuming a linear relationship.

## 3. Results

### 3.1. Comparision of This Study with Previous Work

**Figure 6:**
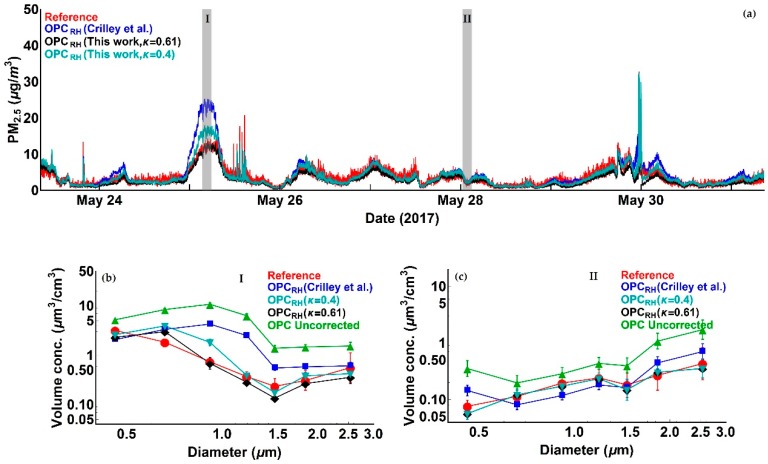
Figures illustrating Alphasense OPC-N2 measurements in comparison with reference data. (**a**) Time series of Alphasense OPC-N2 PM_2.5_ measurements after the application of the correction factor [16] and the correction algorithm (this work) assuming a κ value of 0.4 (Cyan) and 0.61 (Black) in comparison with reference data; (**b**) Size distribution of Alphasense OPC-N2 volume concentration data for all size bins contributing to PM_2.5_ before and after the application of the correction factor [16] and the correction algorithm (this work) assuming a κ value of 0.4 (Cyan) and 0.61 (Black) in comparison with reference data for period I (25 May 03:18:00 UTC–25 May 05:48:00 UTC); (**c**) as (**b**) except for period II (28 May 00:31:00 UTC–28 May 03:01:00 UTC).

As outlined in the Introduction, a recent study [16] has derived a correction factor to account for the relative humidity effect on PM measurement as

(9) $$C=1+\frac{\frac{\kappa }{1.65}}{-1+\frac{1}{a_{w}}},$$

(10) $$PM\left(Corrected\right)=\frac{PM\left(Raw\right)}{C},$$

where *a_w_* is the water activity, defined as *RH*/100, and the statistically derived κ value for their data is in the 0.38–0.41 range [16]. For clarity, in this paper, we took 0.4 as the κ value for the correction presented in [16]. PM_2.5_ measurements from the Alphasense OPC-N2 between 23 May and 31 May 2017, after the application of the correction factor proposed in [16], and the correction algorithm presented in this study, in comparison with the reference data, are presented in Figure 6. To compare our correction approach to the one presented in [16], Figure 6 also presents our correction applied using two different κ values: 0.4, consistent with [16], and 0.61, as discussed in Section 2.3.3. As there is no information regarding the efflorescence point of the compound with κ = 0.4, we have assumed it to be the same as Ammonium Sulphate (*RH* = 35%). Also shown are particle volumes as functions of particle size for two selected periods. As previously mentioned, the size distribution for the Crilley et al. correction factor [16] has been inferred by applying a single correction factor to uncorrected OPC volume concentration data.

It is apparent from Figure 6 that, in most of cases, both correction methods significantly improve the Alphasense OPC-N2 measurements when compared to reference data. There are, however, periods where the correction factor introduced in [16] performs significantly less well (i.e., 24 May, 25 May and 30 May), while the correction algorithm proposed in this work, for both κ values, performs better. The discrepancy between the two correction methods can be explained by considering the changes in the particle size distributions for the different periods. The approach in [16] can be thought of as a sensitivity correction which is independent of particle size, its application leading to a uniform reduction in particle volume concentration and in turn PM. When the particle size distribution of the Alphasense OPC-N2 differs from the reference by a constant factor (Figure 6c) the single value correction approach of [16] works well. In contrast, when the particle size distribution measured by Alphasense OPC-N2 exhibits different profile from the reference (Figure 6b), this simple approach fails. The correction method presented in this work, however, shows a high level of agreement with the reference data for both periods. Moreover, when using a κ value of 0.61 not only we achieve better agreement in particle size distribution with reference data, but we also match the reference mass concentration values. It should be noted that both in this study and that of Crilley et al., the instruments used were the same (Alphasense OPC-N2) covering the same particle size range. As indicated in Table 1, the Alphasense OPC-N2 does not measure particles with a diameter <0.38 μm. Crilley et al. derived their kappa values by comparing their OPC measurements with TEOM measurements which capture particles below the size limit of the Alphasense OPC-N2. By doing this, their retrieved kappa value not only corrects for the *RH* effect but also compensates for the small particles. For these reasons, all the *RH* corrected data presented hereafter are calculated assuming κ = 0.61, unless otherwise stated.

**Figure 7:**
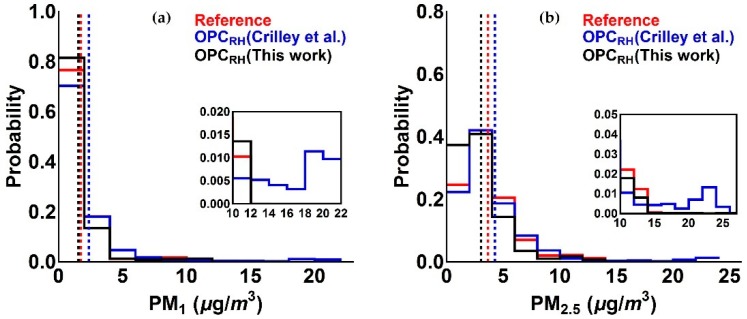
Probability distribution plot of Alphasense OPC-N2 measurement, after the application of the correction factor [16] and the correction algorithm (this work), in comparison with reference data for (**a**) PM_1_ and (**b**) PM_2.5_. The dashed lines represent the mean of PM values in each case. The inserts figures show PM probabilities at higher values on expanded scales (see text).

Probability distribution plots of PM_1_ and PM_2.5_ measurements for corrected Alphasense OPC-N2 and reference data are presented in Figure 7. The figure shows that while the distributions for all three sets of measurements are broadly similar, the Crilley et al. correction overestimates the number of high aerosol events for both PM_1_ and PM_2.5_. This is reflected in the averages in each case (see Table 3).

### 3.2. Statistical Evaluation of the RH Algorithm

**Figure 8:**
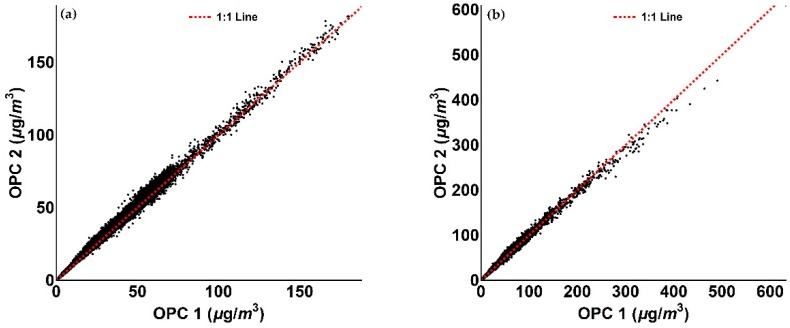
Figures illustrating the reproducibility of the two Alphasense OPC-N2. (**a**) Scatter plot of OPC 1 and OPC 2 PM_1_ measurement relative to the period between 17 December 2017 and 16 January 2018; (**b**) OPC 1 and OPC 2 PM_2.5_ measurement scatter plot of OPC 1 and OPC 2 PM_1_ measurement relative to the period between 17 December 2017 and 16 January 2018.

The reproducibility of the Alphasense instruments was evaluated by co-locating two OPC-N2 units in the period 17 December 2017–16 January 2018. Comparison of the two sensors are presented in Table 4 and Figure 8. Given the high level of reproducibility, the rest of this work will present measurements only from a single OPC (OPC 1).

**Figure 9:**
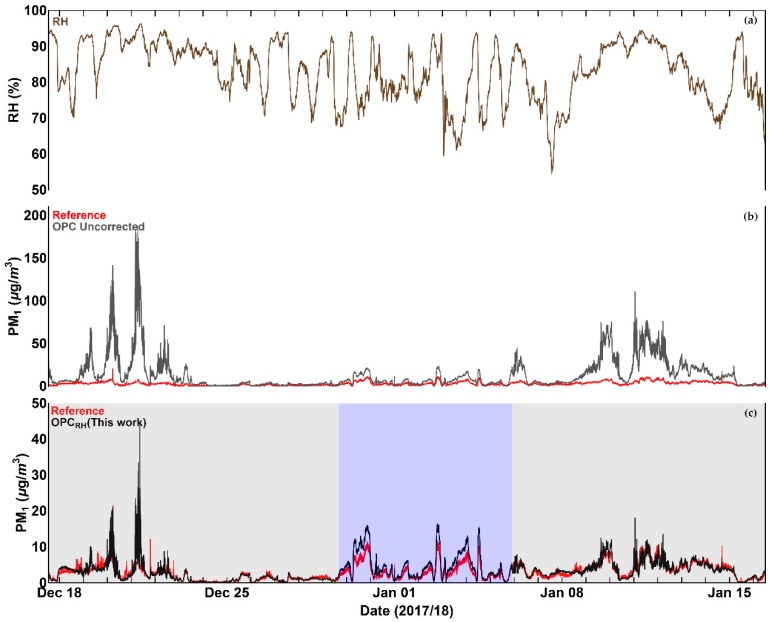
Time series plots of (**a**) Relative Humidity, (**b**) OPC PM_1_ measurement in comparison with reference data before the application of the correction algorithm, and (**c**) OPC PM_1_ measurement in comparison with reference after the application of the correction algorithm. The blue shaded area denotes a period during which the corrected PM values show a systematic overestimation compared to with reference data. More details are given in the text.

**Figure 10:**
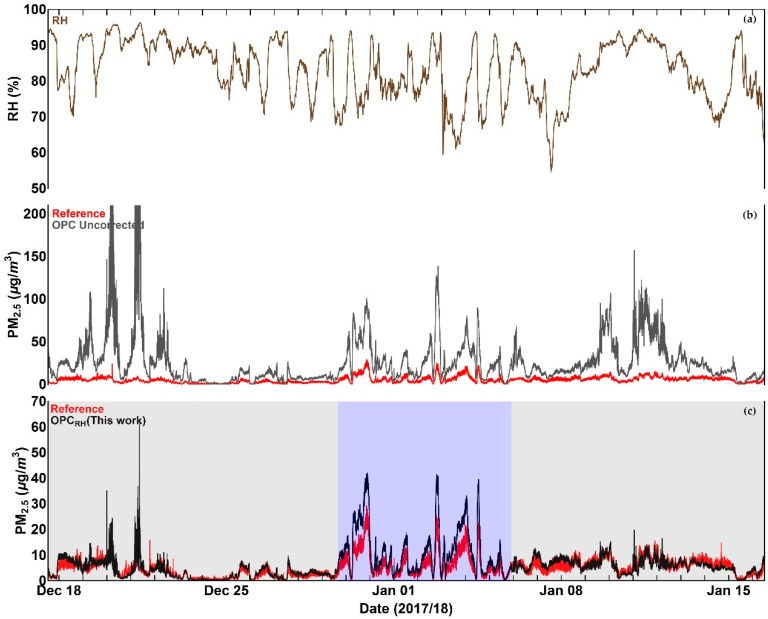
Time series plots of (**a**) Relative Humidity, (**b**) OPC PM_2.5_ measurement in comparison with reference data before the application of the correction algorithm, and (**c**) OPC PM_2.5_ measurement in comparison with reference after the application of the correction algorithm. The blue shaded area denotes a period during which the corrected PM values show a systematic overestimation compared to with reference data. More details are given in the text.

Time series of OPC data in comparison with the reference measurements, before and after the application of the correction algorithm, are shown for PM_1_ and PM_2.5_ in Figure 9 and Figure 10 respectively.

**Figure 11:**
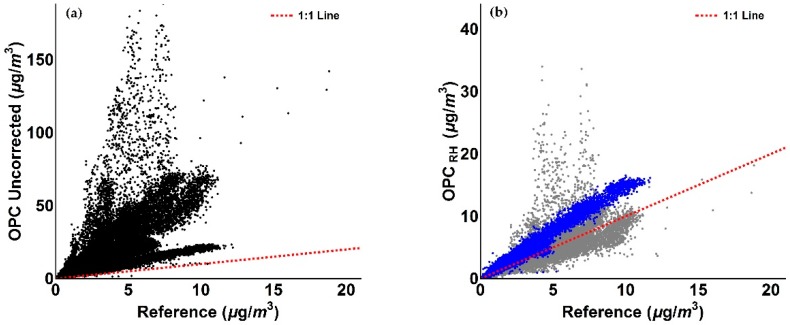
Figures illustrating the comparison of OPC measurements with reference data for PM_1_ values. (**a**) Scatter plot of reference and uncorrected OPC PM_1_ measurements relative to the period between 17 December 2017 and 16 January 2018; (**b**) Scatter plot of reference and OPC *RH*-corrected PM_1_ measurements relative to the period between 17 December 2017 and 16 January 2018. The colour scheme reflects the blue and grey shaded areas in Figure 9c.

**Figure 12:**
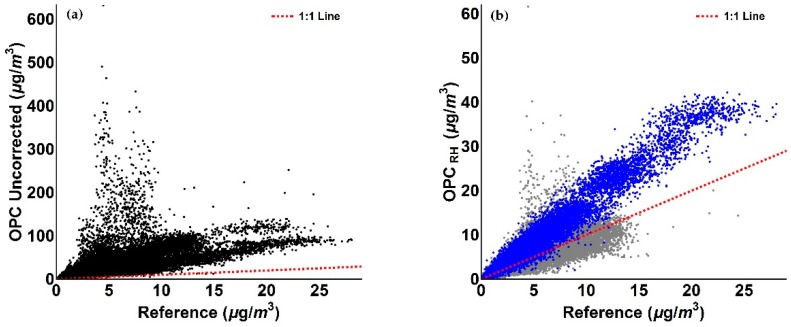
Figures illustrating the comparison of OPC measurements with reference data for PM_2.5_ values. (**a**) Scatter plot of reference and uncorrected OPC PM_2.5_ measurements relative to the period between 17 December 2017 and 16 January 2018; (**b**) Scatter plot of reference and OPC *RH*-corrected PM_1_ measurements relative to the period between 17 December 2017 and 16 January 2018. The colour scheme reflects the blue and grey shaded areas in Figure 10c.

**Figure 13:**
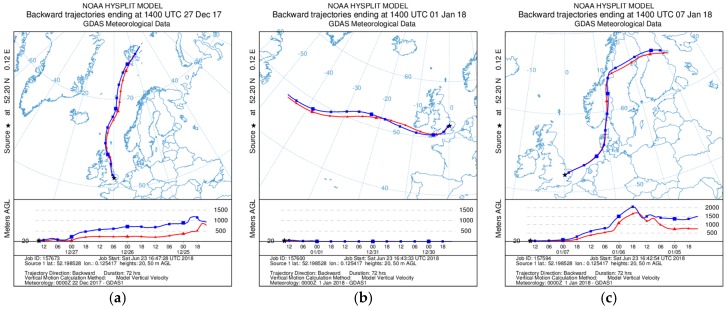
Ready Hysplit air mass trajectories plots ending in Cambridge (UK). (**a**) 27 December 2017; (**b**) 1 January 2018; (**c**) 7 January 2018. The blue and red lines correspond to trajectories at 20 m and 50 m above ground level, respectively.

Inspection of Figure 9, Figure 10, Figure 11 and Figure 12 indicates that a substantial proportion of the *RH* induced peaks in the uncorrected OPC measurements are accounted for when the *RH* algorithm described in this work is applied, and that the level of agreement between the corrected OPCs measurements and reference data is substantially improved (see Table 5). It should be noted, however, that there remains a systematic overestimation in both PM_1_ and PM_2.5_ between 29 December 2017 and 5 January 2018 (the blue shaded area in Figure 9c and Figure 10c), suggesting that the particles are more hygroscopic (i.e., absorbing more water) during this period. To further investigate this, we ran the Ready Hysplit model developed by NOAA to determine the trajectories of air masses for the December–January period, as shown in Figure 13.

**Figure 14:**
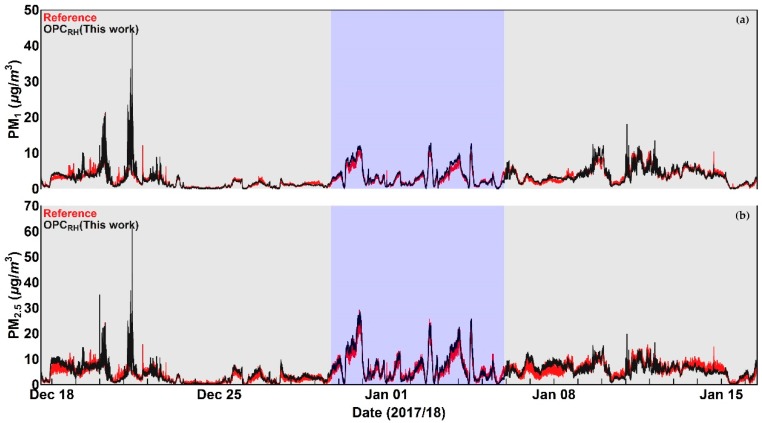
Time series plots of OPC PM measurements in comparison with reference data after the application of the correction algorithm assuming Ammonium Sulphate (grey shaded area) and Sodium Chloride (blue shaded area) as unique particle chemical species for: (**a**) PM_1_; (**b**) PM_2.5_.

The trajectories show clear changes of air mass origin with the period in early January originating in the east Atlantic and remaining in the boundary layer throughout (Figure 13b), while the periods in Figure 13a,c originated from the Arctic mid-troposphere. It is therefore not unreasonable that there are differences in PM composition, and in fact, the early January period PM hygroscopicity is consistent with that of sodium chloride (NaCl). The sodium chloride κ-value is $\kappa_{NaCl}=1.28$ [25], and its efflorescence point is at *RH* = 45.5 ± 0.6% [28], i.e., more hygroscopic than ammonium sulphate. Applying the correction algorithm to the period 29 December 2017 and 5 January 2018 but changing the chemical component from ammonium sulphate to sodium chloride gives the results shown in Figure 14.

**Figure 15:**
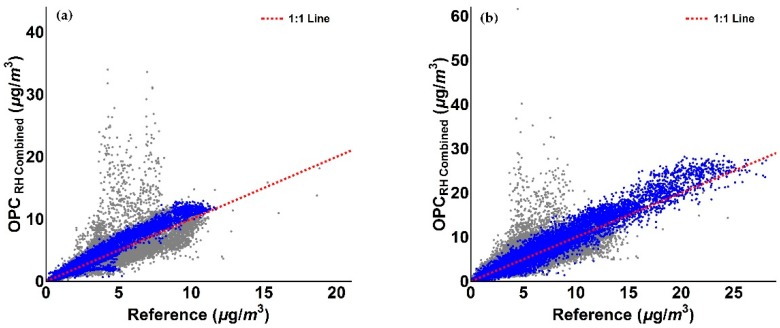
Figures illustrating the comparison of *RH* corrected PM measurements assuming Ammonium Sulphate (grey points) and Sodium Chloride (blue points) as unique chemical species for PM for the periods discussed above. (**a**) PM_1_; (**b**) PM_2.5_.

We observe from Figure 14 that by assuming NaCl as sole chemical component for this specific period (blue shaded area), the agreement of the corrected PM measurements with reference data has substantially improved. This also results in an improvement in correlation, as detailed in Figure 15.

As evident from Figure 15, the measurements associated with the period where NaCl is assumed to be the sole chemical species (blue) show an improved agreement with the 1:1 line compared to assuming ammonium sulphate as the sole chemical species (cf. Figure 11b and Figure 12b). 

The values reported in Table 5 confirm the improvements achieved by the correction method. When we assumed ammonium sulphate as single particle component for the 17 December 2017–16 January 2018 period, the mean value of OPC measurements was improved from a factor of 4.45 before correction to 1.15 after correction, as well as the gradient from a factor of 5.25 to a factor of 1.15 and the *R*^2^ from 0.42 to 0.73, before and after the application of the correction algorithm, respectively. When sodium chloride was assumed for the 29 December 2017–5 January 2018 period (blue shaded area in Figure 14) with ammonium sulphate elsewhere, the mean value of OPC measurements was improved from a factor of 4.45 before correction to 1.06 after correction, as well as the gradient from a factor of 5.25 to a factor of 1.05 and the *R*^2^ from 0.42 to 0.75, before and after the application of the correction algorithm, respectively. Mean values of measurements, SD, RMSE, gradient and coefficient of determination *R*^2^ values relative to PM_2.5_ data are presented in Table 6. As previously observed for PM_1_ data, values in Table 6 confirm the improvement of the *RH* algorithm for OPC PM_2.5_ measurements. Specifically, when ammonium sulphate was taken as the single particle component for the 17 December 2017–16 January 2018 period, the mean value of OPC measurements was improved from a factor of 5.10 before correction to 1.26 after correction, as well as the gradient from a factor of 4.59 to a factor of 1.43 and the *R*^2^ from 0.34 to 0.75, before and after the application of the correction algorithm, respectively. Again, when sodium chloride was assumed as sole particle component for the 29 December 2017–5 January 2018 period (blue shaded area in Figure 14) and ammonium sulphate elsewhere, the mean value of OPC measurements was improved from a factor of 5.10 before correction to 1.06 after correction, as well as the gradient from a factor of 4.59 to a factor of 1.01 and the *R*^2^ from 0.34 to 0.78, before and after the application of the correction algorithm, respectively.

## 4. Conclusions

Prior works have illustrated how low-cost portable sensors can be used to measure concentrations of particulate mass [11,12,29]. A recent study has focused on the effects of relative humidity on measurements [16]. However, unlike that study which proposed a correction for PM which effectively implies a uniform change in particle number at all sizes, in this study we have introduced an algorithm to correct for the changes in individual particle size due to water uptake under high *RH* conditions which reflects the hygroscopic properties of real world particles. The algorithm provides an adjusted particle size distribution which is not a simple scaling, and adjusted PM values. In this paper we have used measurements from a low cost OPC (Alphasense OPC-N2) and a reference OPC (Palas Fidas 200 S) over a six-week period (23 May 2017–31 May 2017 and 17 December 2017–16 January 2018). Under the assumption that urban particles consist of ammonium sulphate, we applied the correction algorithm to the Alphasense OPC-N2 measurements. The results showed that the overall level of agreement between the corrected OPC measurements and reference data was substantially improved (reduced overestimation from a factor of 5.25 to 1.15 for PM_1_ and from a factor of 4.59 to 1.43 for PM_2.5_). Nonetheless, there was a period where the corrected PM measurements still consistently overestimated the reference observations to a small degree. We show this event corresponds to a change in air mass origin consistent with a change in particle hygroscopicity. Our analysis showed that the particle hygroscopicity during this overestimation period was consistent with that of sodium chloride (NaCl). By assuming sodium chloride during the overestimation period and ammonium sulphate elsewhere, the corrected Alphasense OPC-N2 measurements improved further when compared to reference data. The results shown in this paper extend those already present in literature on the capacity of low-cost sensors to give reliable ambient PM readings when an appropriate correction is applied. While this work was performed using the instrument characteristics of an Alphasense OPC-N2, this algorithm is independent of sensor type and can be readily adapted to other size speciated particle counters and different environments. Finally, we note that the correction algorithm presented in this work not only is flexible to changes in particle chemical composition but also leads to the possibility of particle chemical speciation using low-cost sensors.

## Figures and Tables

**Table 1:** Summary of Alphasense OPC-N2 operational settings [18].

	Alphasense OPC-N2
Sampling time (s)	1.4
Size range (µm)	0.38–17.0
Number of size bins	16
Flow rate (L/min)	1.2
Data storage (GB)	16
Weight (Kg)	0.105
Dimensions H·W·D (mm)	63.5 × 75 × 60
Temperature range (°C)	−10 to +50

**Table 2:** Summary of Palas Fidas 200 operational settings [19].

	Palas Fidas 200 S
Sampling time (s)	60 (average)
Size range (µm)	0.18–18.0
Number of size bins	64
Flow rate (L/min)	4.8
Data storage (GB)	4
Weight (Kg)	60
Dimensions H·W·D (mm)	1810 × 600 × 400
Temperature range (°C)	−20 to +50

**Table 3:** Average PM values for the corrected OPC and reference measurements in Figure 6.

	Reference	Crilley et al.	This Work
PM_1_ (μg/m^3^)	1.74	2.36	1.55
PM_2.5_ (μg/m^3^)	3.64	4.25	3.03

**Table 4:** Correlation values for Alphasense OPC-N2 sensors used during this study.

OPC	PM_1_		PM_2.5_	
	Gradient	*R* ^2^	Gradient	*R* ^2^
1	1.00	1.00	1.00	1.00
2	1.03	0.99	0.99	0.99

**Table 5:** Statistical parameters for PM_1_ measurement of reference, uncorrected OPC, and OPC after the application of the *RH* algorithm.

PM_1_	Reference	OPC (Uncorrected)	OPC (*RH* Corrected)	OPC (*RH* Combined)
Mean (µg/m^3^)	3.02	13.45	3.46	3.20
SD (µg/m^3^)	2.25	18.24	3.03	2.72
RMSE (µg/m^3^)	N.A.	19.84	1.66	1.37
Gradient	1.00	5.25	1.15	1.05
*R* ^2^	1.00	0.42	0.73	0.75
Number of points	43,000	43,000	43,000	43,000

**Table 6:** Statistical parameters for PM_2.5_ measurement of reference, uncorrected OPC, and OPC after the application of the *RH* algorithm.

PM_2.5_	Reference	OPC (Uncorrected)	OPC (*RH* Corrected)	OPC (*RH* Combined)
Mean (µg/m^3^)	5.12	26.10	6.47	5.44
SD (µg/m^3^)	3.69	28.85	6.07	4.21
RMSE (µg/m^3^)	N.A.	35.78	5.91	3.74
Gradient	1.00	4.59	1.43	1.01
*R* ^2^	1.00	0.34	0.75	0.78
Number of points	43,000	43,000	43,000	43,000
